# Modern Strategies for Brucellosis Vaccination: From Traditional Approaches to Innovative Platforms

**DOI:** 10.3390/vaccines14050409

**Published:** 2026-05-01

**Authors:** Nurika Assanzhanova, Kuandyk Zhugunissov, Olga Chervyakova, Sholpan Ryskeldinova, Nurlan Akmyrzayev, Aigerim Sagymbayeva, Yeldos Myrzakhmetov, Aigerim Mailybayeva

**Affiliations:** Research Institute for Biological Safety Problems, National Holding “QazBioPharm”, Gvardeiskiy 080409, Kazakhstan; n.assanzhanova@biosafety.kz (N.A.); k.zhugunisov@biosafety.kz (K.Z.); o.chervyakova@biosafety.kz (O.C.); sh.ryskeldinova@biosafety.kz (S.R.); a.sagymbayeva@biosafety.kz (A.S.); y.myrzakhmetov@biosafety.kz (Y.M.)

**Keywords:** *Brucella*, vaccines, *Brucella melitensis*, *Brucella abortus*, vector vaccines, subunit vaccines, DNA vaccines, human vaccines, DIVA, One Health

## Abstract

Brucellosis remains one of the most widespread zoonotic infections worldwide, causing serious veterinary, medical, and socio-economic consequences. The disease, caused by bacteria of the genus *Brucella*, affects a wide range of domestic and wild animals as well as humans, with global incidence potentially reaching 1.6–2.1 million new cases annually. The most effective approach to combating brucellosis is specific prevention through vaccination. Therefore, we conducted this review to summarize data from existing studies on modern strategies for brucellosis vaccination, types of vaccine platforms, their efficacy, safety, and applicability in veterinary and human medicine. We searched databases including PubMed, Scopus, and Web of Science to identify relevant scientific articles in English published from 1990 to 2025. The aim of this work is to conduct a systematic analysis of modern brucellosis vaccination strategies in livestock and humans, as well as to evaluate the prospects of new vaccine platforms. The review examines live attenuated, inactivated, subunit, vector, and DNA vaccines, as well as their immunological mechanisms of action, advantages, and limitations of application. This information allows for a better understanding of the mechanisms of protective immunity formation and challenges related to DIVA diagnostics (Differentiating Infected from Vaccinated Animals). The “One Health” concept demonstrated the interconnection between human, animal, and environmental factors, emphasizing the need for an interdisciplinary approach to brucellosis monitoring, prevention, and control. Vector vaccines based on influenza virus (Flu-BA), developed in Kazakhstan, have shown high promise, combining immunogenicity, protective efficacy, and a favorable safety profile. Promising directions remain mRNA vaccines, nanoparticles, CRISPR/Cas9 technologies, and mucosal vaccines.

## 1. Introduction

Brucellosis is a chronic zoonotic infectious disease that represents a serious veterinary, medical, and socio-economic problem in many countries worldwide. The infection is caused by bacteria of the genus *Brucella*, capable of inducing systemic febrile disease in animals and humans. The spread of brucellosis leads to significant economic losses in livestock production, reduced productivity, and restrictions on international trade in animals and animal products.

The zoonotic nature of the disease determines a close relationship between the epizootic process in livestock populations and human morbidity. Consequently, brucellosis has a substantial impact on food security systems and population livelihoods [1,2,3]. Modern epidemiological data indicate that the actual global incidence significantly exceeds previously published estimates and may reach 1.6–2.1 million new human cases annually [4,5].

**Figure 1:**
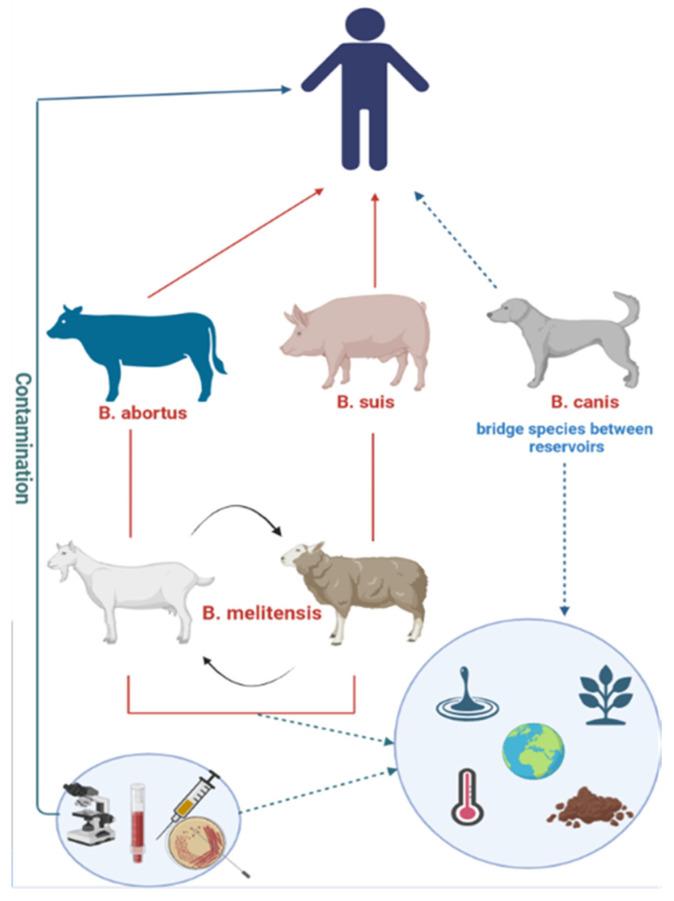
Epidemiological relationships between the main reservoirs of *Brucella* bacteria in animals and mechanisms of their transmission to humans. Red solid arrows indicate direct zoonotic transmission from infected animals to humans through direct contact. Dashed arrows represent indirect transmission via environmental contamination (soil, water, vegetation). The diagram illustrates the complex interplay between animal reservoirs (cattle, small ruminants, pigs), environmental factors, and human infection routes, emphasizing the importance of the “One Health” approach in brucellosis control.

In livestock, brucellosis is accompanied by severe reproductive disorders, including late-term abortions, intrauterine fetal death, infertility, and reduced productivity [6,7]. These consequences are particularly significant for low- and middle-income countries where agriculture plays a key role in the economy. Simultaneously, the infection poses a serious threat to public health due to increasing morbidity in both animals and humans [8,9]. Human infection occurs primarily through direct contact with infected animals or consumption of contaminated dairy products (Figure 1) [9].

The environment (soil, water, plants) serves as a long-term reservoir for the pathogen. Laboratory diagnostics (microbiological, serological, and molecular methods) play a key role in detecting and controlling infection.

Brucellosis can also cause flu-like symptoms in humans (e.g., resembling malaria, typhoid fever, streptococcal infections, and rheumatoid arthritis). Clinically, it cannot be distinguished from other flu-like diseases, except for rheumatoid arthritis [10]. As a result, physicians face difficulties in treating acute human brucellosis [11].

Identifying occupational and behavioral risk factors is essential for developing effective preventive measures, including improving sanitation and hygiene practices, enhancing working conditions, and implementing animal vaccination programs. It is estimated that approximately 3.5 billion people worldwide live in areas with potential risk of brucellosis infection. The disease is accompanied by significant economic losses related to treatment costs, reduced work capacity, and decreased livestock productivity [12,13].

The most effective way to combat brucellosis is specific prevention through vaccination. This method is considered economically feasible and particularly relevant for countries with high endemicity. However, the choice of vaccination strategy must consider the epizootiological situation, type of livestock, socio-economic conditions, and other factors.

The aim of this review is to systematically analyze modern strategies for brucellosis vaccination in livestock and humans, with emphasis on comparative evaluation of the efficacy and safety of existing vaccine platforms. This paper examines live attenuated, inactivated, subunit, vector, and DNA vaccines, their immunological mechanisms of action, advantages, and limitations of application.

## 2. Literature Search Methodology

To prepare this review, a systematic search of scientific literature on the development and application of vaccines against brucellosis in animals and humans was conducted.

The search for publications was carried out in international scientific databases such as PubMed, Scopus, and Web of Science. Additionally, materials from international organizations such as the World Health Organization (WHO) and World Organisation for Animal Health (WOAH), as well as references from key publications, were used.

The search covered publications issued from 1990 to 2025. Primary attention was given to articles published in English in peer-reviewed scientific journals. The following keywords and their combinations were used for the literature search: *Brucella* vaccine, Brucellosis vaccination, live attenuated *Brucella* vaccine, *Brucella* vector vaccine, *Brucella* DNA vaccine, *Brucella* subunit vaccine, DIVA vaccine for brucellosis, *Brucella melitensis* vaccine, and *Brucella abortus* vaccine.

Methodological note: While the primary search was limited to PubMed, Scopus, and Web of Science to ensure methodological rigor, we recognize that this may have underrepresented regionally published studies and grey literature. Official WHO/WOAH reports and verified national data were incorporated to enhance contextual relevance. Future updates may expand the search to specialized veterinary databases (e.g., CAB Abstracts, AGRIS) when resources permit.

At the first stage, more than 300 publications were identified. After assessing relevance and study quality, the most significant and highly cited works were included in the review, covering various types of vaccines, including live attenuated, inactivated, subunit, DNA, and vector vaccines.

Methodological note on study selection. As this is a narrative review, the final inclusion of studies was guided by methodological quality, direct relevance to vaccine platform evaluation, and contribution to understanding immunological mechanisms and protective efficacy against *Brucella* spp. While citation frequency was considered as one indicator of scientific impact, it was not the sole selection criterion. Efforts were made to include recent publications (2020–2025) and studies from endemic regions, even when citation counts were lower, to ensure contemporary and geographically balanced coverage. Nevertheless, we acknowledge that some relevant but less-visible studies may not have been captured, and future updates of this review may adopt more formal systematic review methodologies to further enhance comprehensiveness.

Note on evidence distribution. The current inclusion criteria reflect the existing evidence landscape, in which brucellosis vaccine research is predominantly conducted in animal models and preclinical settings due to the absence of licensed human vaccines and ethical constraints on clinical trials. While this ensures methodological rigor and reproducibility, it inherently limits the direct applicability of conclusions to human vaccination strategies. Future updates of this review will incorporate emerging human clinical and phase I/II trial data as they become available.

Methodological note on review type and synthesis approach. This work is a narrative review incorporating systematic literature search elements. Due to substantial heterogeneity across included studies—in terms of *Brucella* species (*B. abortus*, *B. melitensis*, *B. suis*), vaccine platforms (live attenuated, subunit, vector, DNA), host species (cattle, sheep, goats, mice, guinea pigs), outcome measures (antibody titers, IFN-γ levels, protection rates), and study designs—quantitative meta-analysis with pooled statistical estimates was not feasible. Consequently, conclusions are based on qualitative synthesis of evidence, allowing contextual interpretation of immunological mechanisms, safety profiles, and translational potential. Readers should interpret findings as representative of current knowledge rather than as statistically pooled effect sizes. Future updates of this review may adopt more formal systematic review methodologies with meta-analytic components as the evidence base expands.

## 3. Prevalence of Brucellosis in Animals and Humans

Brucellosis represents one of the most widespread zoonotic infections worldwide, caused by bacteria of the genus *Brucella*, affecting a wide range of domestic and wild animals, including cattle, small ruminants, pigs, dogs, and other mammals [14,15].

The highest prevalence of brucellosis is observed in countries of the Mediterranean region, the Middle East, Central Asia, and Africa. Hyperendemic territories include Iran, Kyrgyzstan, Tajikistan, Kazakhstan, Azerbaijan, Turkmenistan, Armenia, and Uzbekistan [16,17]. In low- and middle-income countries, the disease remains a serious medical-veterinary problem due to high livestock density, insufficient vaccination coverage, and limited capacity for epizootic control.

Among these hyperendemic territories, the Republic of Kazakhstan serves as a representative example of the persistent brucellosis burden in Central Asia. Brucellosis has remained an endemic zoonotic infection in Kazakhstan since the 1930s, posing a significant challenge for veterinary and public health systems. Annually, more than 1300 human cases are registered (incidence rate: 7.6 per 100,000 population), classifying the country as a high-burden territory. Seropositivity in livestock remains at approximately 0.6% in cattle and 0.4% in small ruminants [18]. The epizootic process exhibits a pronounced focal pattern: during 2020–2024, 1314 out of 2,460,956 tested small ruminants reacted positively (0.05%), with up to 78.6% of detected cases concentrated in certain regions [19,20]. Key risk factors include consumption of unpasteurized dairy products and direct contact with aborted materials, exacerbated by the predominance of smallholder farms that account for a substantial proportion of the national livestock population [21].

In contrast, several economically developed countries have achieved elimination of brucellosis among livestock through the implementation of comprehensive control programs, including mandatory vaccination, serological monitoring, culling of infected animals, and strict veterinary surveillance [22,23,24,25].

The prevalence of infection in humans directly correlates with the epizootic situation in animals, emphasizing the importance of the “One Health” concept. The main risk factors for infection include contact with infected animals, consumption of unpasteurized dairy products, and occupational activities in the livestock sector.

## 4. Pathogenesis and Immune Response

Understanding the mechanisms of interaction between the pathogen and the host organism, as well as the molecular basis of brucellosis pathogenesis, is essential for developing rationally designed vaccines. Like other pathogenic intracellular bacteria, representatives of the genus *Brucella* implement the infectious process through sequential stages: adhesion to host cells, invasion, intracellular establishment, and subsequent spread throughout the organism [26]. One of the key features of brucellosis infection is its ability to proceed in a so-called “stealth mode,” ensuring pathogen evasion from recognition by the innate immune system [27,28]. This strategy promotes the formation of persistent infection and chronicity of the pathological process.

The immune response against *Brucella* includes both innate and adaptive defense mechanisms, with Th1-mediated cellular immunity playing a key role. IFN-γ production by CD4^+^ and CD8^+^ T-lymphocytes activates bactericidal functions of macrophages and promotes elimination of intracellular pathogens. Understanding the features of pathogenesis and *Brucella* interaction with the host immune system is an important aspect for developing effective and safe vaccines against brucellosis.

## 5. Brucellosis Vaccine Development

Vaccination is a promising method for controlling the spread of brucellosis, particularly in livestock. Various vaccines have been developed for animals, but their efficacy varies. However, currently no vaccine for humans exists.

Currently, there are no officially approved vaccines against brucellosis in humans. It should be noted that most evidence regarding immunogenicity and protective efficacy of brucellosis vaccines derives from experimental animal models. While these data provide valuable mechanistic insights, direct extrapolation to human applications requires caution due to species-specific differences in immune response and pathogenesis. In this review, we explicitly distinguish findings supported by clinical evidence in humans from those based on preclinical or veterinary studies, using formulations such as “translational potential,” “data extrapolation,” and “limited human evidence base” where appropriate. The lack of available vaccines hinders the control of this disease in humans [29]. Therefore, controlling brucellosis in animals is the most effective approach to preventing human infection [30]. Below are different types of brucellosis vaccines and their efficacy.

### 5.1. Live Attenuated Vaccines

In recent decades, the most effective method for combating brucellosis in animals has been the use of live attenuated vaccines. Live attenuated vaccines are the most effective method for controlling animal brucellosis [31]. Live attenuated vaccines are easily reproducible, use accessible bacterial materials with simple preparation technology, are more effective, and induce strong immunity through humoral and cellular responses in animals. Live attenuated vaccines include those from *B. abortus* S19 and *B. abortus* RB51 strains intended for cattle, *B. melitensis* Rev1 strain for small ruminants, and S2 *B. suis* strain for swine brucellosis [32]. Each is known to be approximately 70% effective and is applied successfully; however, due to residual pathogenicity that can cause disease, they are unsuitable for use in humans [33]. These vaccines are successfully applied in Russia, France, and other Western European countries.

Studies of the efficacy of these vaccines were conducted in experimental animals. During the research, full protection against wild-type (WT) bacteria was established. Since stimulating antibody production complicates serodiagnosis in detecting infected animals, according to J. Ko, E. M. Dorneles, and other authors, an “ideal” brucellosis vaccine should contain live bacteria capable of generating a powerful Th1 cellular response in the host, induce long-term protective effect after a single vaccine dose without side effects, contain attenuated *Brucella* strains that do not cause disease or persistent infection in animals, be stable and not revert to virulent form either in vivo or in vitro, not lead to seroconversion upon revaccination, be safe for humans in case of accidental infection during animal vaccination, be accessible for mass application, and be simple in production and use [34,35].

The attenuated *B. melitensis* Rev-1 strain differs in high residual virulence and immunogenicity. One of the disadvantages of *B. melitensis* vaccines is their antibiotic resistance to rifampicin and streptomycin, which are the drugs of choice for treating brucellosis. Moreover, using this vaccine creates a risk of transmitting antibiotic resistance genes to wild *Brucella* strains. When vaccine strains of *Brucella* with smooth colony type (S-form), such as *B. abortus* S19 and *B. melitensis* Rev1, are introduced into the organism, an antibody response to the O-polysaccharide (OPS) of lipopolysaccharide (LPS) in their cell walls is formed. Consequently, serodiagnosis of brucellosis based on detecting diagnostic titers of anti-LPS antibodies will subsequently be uninformative. This preparation has a negative indicator for the ability to differentiate vaccinated from infected animals (Differentiation of Infected from Vaccinated Animals (DIVA)). This indicator is considered by modern researchers as an important criterion for the suitability of developed vaccines [36].

### 5.2. Inactivated Vaccines

Inactivated anti-brucellosis vaccines generally demonstrate low protective efficacy and require the use of adjuvants and administration of multiple booster doses. In a study by Won-Kyong Kim et al. (2019), it was shown that using *B. abortus* cells lysed by GI24 (a fragment of porcine myeloid antimicrobial peptide) for intradermal immunization of goats stimulated elevated levels of IL-4, TNF-α, IFN-γ, and anti-LPS antibodies compared to control animals, and upon subsequent subconjunctival challenge with virulent *B. abortus* 544 strain, three of five goats were protected from infection [37].

In the early stages of brucellosis vaccine development, primary research focus was directed toward investigating cellular components of inactivated bacteria, such as outer membrane proteins, cell wall fractions, whole killed cells, and periplasmic proteins [38,39,40]. Although these foundational studies established key principles in vaccine prophylaxis, contemporary scientific priorities have shifted toward optimizing antigen delivery systems and adjuvant formulations designed to enhance immunogenicity and prolong protective immunity [41,42]. Data from recent systematic reviews indicate that incorporating nanoparticles as adjuvants into modern inactivated vaccines significantly strengthens the Th1-mediated immune response compared to traditional formulations, thereby overcoming historical limitations associated with short-term protection and the requirement for high-dose administration [41,43].

Regarding inactivated vaccines against brucellosis, they were considered as a safer alternative to live *Brucella melitensis* Rev.1 and *Brucella abortus* S19 vaccine strains. Such preparations are prepared by inactivating bacterial cells (most commonly using formalin or heat treatment) and their subsequent administration with mineral or oil adjuvants. Inactivated vaccines demonstrated an acceptable safety level and did not cause prolonged bacteriocarriage, making them convenient for use in farms where live vaccines were undesirable. However, their disadvantages remained relatively low and short-term immunity, the need for multiple revaccinations, and high production costs. Despite this, inactivated preparations found application in brucellosis prevention and control programs in sheep and goats in several countries, especially in combination with serological monitoring and strict veterinary-sanitary measures.

### 5.3. Subunit Vaccines

Subunit vaccines are promising vaccine candidates due to their safety profile, clearly defined non-infectious nature, inability to revert to virulent strains, non-viability unlike attenuated vaccines, ability to induce high antibody levels, and capacity for manipulation to maximize desired activity. These vaccines incorporate recombinant highly conserved proteins that can target multiple *Brucella* species [44]. Poor antigenicity, instability, and short half-life of recombinant subunit antigens are the main obstacles in developing an effective subunit vaccine against brucellosis [45].

Creating strong and prolonged immunity through artificial immunization is determined by the animal’s organism condition, quantity and quality of antigen in the preparation, method of administration, and correct choice and use of non-specific immunogenesis stimulants—adjuvants.

Many studies have demonstrated relatively promising results using subunit vaccines against brucellosis. Although subunit vaccines have the advantage of safety, they require multiple revaccinations and combination of several antigens, adjuvants, and carriers/vectors to induce effective immunity and protection against *Brucella*, which is economically inexpedient [46]. Moreover, most studies have been performed in mouse models, and the immune response observed in mice may not correspond to the level of protection achieved in natural hosts after vaccination. Therefore, further comprehensive studies are needed to discover recombinant vaccines including multiple *Brucella* antigens.

### 5.4. Vector Vaccines

Live vector vaccines, in which *Brucella* is used as an antigen delivery system, are considered a promising tool for presenting both homologous and heterologous antigens. Such genetically modified constructs are capable of inducing antigen-specific T-cell immune responses through intracellular replication in the host organism and synthesis of multiple copies of *Brucella* antigens [47]. Various viral and bacterial vectors, including *Salmonella* spp., *Escherichia coli*, and influenza viruses, are used for expressing *Brucella* proteins.

**Figure 2:**
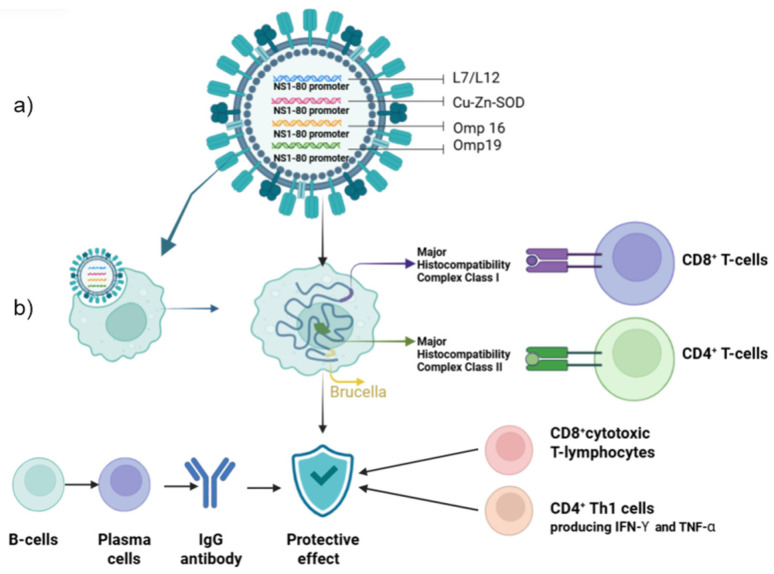
Construction and mechanism of action of the Flu-BA vector vaccine against brucellosis. (**a**) Structure of the recombinant influenza virus expressing *Brucella* antigens (L7/L12, Omp16, Omp19, Cu-Zn-SOD) under the control of the NS1-80 promoter. HA, hemagglutinin; NA, neuraminidase. (**b**) Scheme of immune response induction: the vaccine infects antigen-presenting cells (APCs), leading to intracellular expression of *Brucella* antigens, their presentation via MHC class I and II molecules, activation of CD8+ cytotoxic and CD4+ helper T-lymphocytes, and induction of humoral immunity (IgG antibodies). The combined response results in the development of protective immunity against brucellosis.

In Kazakhstan, a vector vaccine against *Brucella abortus* based on influenza virus (Flu-BA) was developed and registered in 2019 [48,49] (Figure 2). This vaccine became the first registered vector vaccine against brucellosis intended for use in veterinary practice.

Recombinant influenza viruses expressing ribosomal protein L7/L12 and outer membrane protein OMP16 of *Brucella* were constructed as vector vaccines [50]. When administered to cattle, the preparation demonstrated a high safety profile compared to S19 vaccine strain. Revaccination (primary booster) ensured the formation of both humoral and cellular immune responses with development of long-term protective immunity, including in pregnant heifers challenged with *B. abortus* [51,52]. Modification of the vaccine composition by including additional antigens OMP19 and SOD, as well as using Montanide gel as an adjuvant, allowed expanding the protection spectrum and providing effective immune protection in sheep and goats upon experimental challenge with *B. melitensis* [53].

In logical continuation of conducted studies, high safety and efficacy indicators of the Flu-BA vector vaccine in cattle and small ruminants demonstrated the feasibility of further expanding its potential. The obtained results served as scientific justification for improving the vector platform and evaluating the possibility of its application in human medicine.

For this purpose, a tetravalent vaccine formulation, Flu-NS1-80-L7/L12 + Flu-NS1-80-Omp16 + Flu-NS1-80-Omp19 + Flu-NS1-80-Cu-Zn-SOD, was developed, which protects guinea pigs from *B. melitensis* 16M infection at a significant level (*p* < 0.05) [54,55]. Research results showed that the tetravalent construct induces optimal immune response and can be considered as a promising direction for further development of human brucellosis vaccine.

Critical evaluation and comparative context of vector platforms. While the Flu-BA vector platform has demonstrated promising safety and efficacy profiles in preclinical and field studies [48,49,50,51,52,53,54,55], several limitations warrant consideration. However, their practical implementation faces certain limitations. In particular, regulatory frameworks for novel viral vector platforms, including Flu-BA, are still under development, which may complicate their clinical translation and widespread adoption. Finally, a substantial proportion of the available data has been generated under limited experimental conditions, highlighting the need for more extensive independent validation studies.

Comparative analysis with alternative viral vector platforms—including replication-deficient adenoviruses, *Salmonella*-based vectors, and poxvirus systems—indicates that each approach presents distinct trade-offs in cargo capacity, cellular tropism, and manufacturing scalability [56]. The Flu-BA platform offers efficient intracellular antigen expression and robust Th1 polarization, yet its human translational potential remains contingent upon independent, multi-center validation studies and comprehensive immunogenicity assessment across diverse host backgrounds.

### 5.5. DNA Vaccines

DNA vaccines are currently being actively developed. They are capable of providing prolonged antigen expression, inducing both cellular and humoral responses in the organism. DNA vaccines are stable, do not require low-temperature storage, are safe, and can be used for human vaccination [46].

*Brucella* vaccines based on DNA are by nature subunit vaccines and form immune response, as a rule, after multiple administrations [57]. These vaccines are characterized by a high safety profile and potential efficacy in brucellosis prevention, which is due to induction of pronounced cellular immune response, possibility of expressing multiple antigens, presence of immunostimulatory CpG motifs, and simple storage conditions [58]. DNA vaccines contain pathogen gene sequences encoding factors critically important for intracellular survival of *Brucella* spp.

The immunogenicity and protective efficacy of such virulence genes have been demonstrated in laboratory animal experiments. In particular, the two-component regulatory system BvrR/BvrS [59], superoxide dismutase Cu-Zn (SOD), ribosomal protein L7/L12 and *Brucella* lumazine synthase (BLS) [58,60], Omp31 and Omp25 genes of *B. melitensis* [41,60], BCSP31 surface antigen gene [61], SP41 protein [62], as well as ribosomal protein L9 (rL9) [63] have been studied.

DNA vaccines are particularly suitable for pathogens requiring robust cellular immunity. However, comparative data indicate that these platforms typically induce a weaker immune response in humans than in murine models, underscoring the need for further optimization prior to clinical translation [64]. While certain optimization strategies may enhance proinflammatory reactions [43], novel approaches focusing on advanced delivery systems and codon optimization offer promising pathways to improve immunogenicity and translational potential [43].

### 5.6. Limitations of Current Vaccine Platforms

Each brucellosis vaccine platform presents distinct trade-offs between safety, immunogenicity, and practical applicability. Live attenuated vaccines remain the veterinary gold standard due to their robust, long-lasting protection, yet their residual pathogenicity, interference with serodiagnosis (DIVA incompatibility), and unsuitability for human use limit their translational potential. Inactivated preparations offer improved safety profiles but require adjuvants, multiple booster doses, and still yield only short-term, suboptimal immunity. Subunit vaccines eliminate infectious risks and enable precise antigen targeting; however, their inherently low immunogenicity necessitates complex multivalent formulations and advanced delivery systems, with most efficacy data currently restricted to murine models. Viral vector-based platforms, including Flu-BA, have successfully demonstrated the capacity to induce both cellular and humoral immunity and can be adapted to comply with DIVA principles. However, their practical implementation faces certain limitations. In particular, regulatory frameworks for novel viral vector platforms, including Flu-BA, are still under development, which may complicate their clinical translation and widespread adoption. DNA vaccines provide exceptional stability and safety, particularly for human applications, but consistently demonstrate weaker immunogenicity in larger animals and humans compared to rodent models, highlighting the need for optimized delivery and codon adaptation. Crucially, the majority of protective efficacy data across all platforms derive from experimental animal studies, and direct extrapolation to human clinical contexts remains constrained by species-specific immunological differences, ethical limitations on human trials, and the absence of standardized challenge models. These evidence gaps underscore the necessity of dedicated translational research, including humanized models, phase I/II safety trials, and harmonized immunological endpoints, to bridge the current divide between veterinary success and human vaccine development.

## 6. Challenges in Vaccine Development

Despite years of research, brucellosis remains one of the most serious zoonotic infections requiring the creation of safe and effective prophylactic agents for livestock and humans.

The efficacy and duration of immunity formed by existing vaccines also remain a subject of discussion. For reliable protection against *Brucella*, a pronounced cellular Th1-type response accompanied by IFN-γ and TNF-α production is necessary; however, most candidates provide only partial or short-term immune responses. Studied individual *Brucella* antigens (Omp16, Omp19, L7/L12, Cu/Zn-SOD, etc.) exhibit immunogenicity but in monocomponent form do not ensure stable protection, which requires the development of multicomponent vaccine constructs and the use of modern adjuvants. The limited protective efficacy of monocomponent subunit vaccines reflects the biological complexity of *Brucella* pathogenesis: as an intracellular pathogen, *Brucella* evades immune clearance through multiple mechanisms, necessitating coordinated activation of both humoral and Th1-type cellular responses. Consequently, vaccine constructs incorporating multiple conserved antigens (e.g., Omp16 + Omp19 + L7/L12 + Cu/Zn-SOD), combined with appropriate adjuvants or delivery systems, are more likely to induce synergistic immune activation and durable protection. This rationale underpins current efforts to develop multicomponent subunit, vector, and mRNA vaccine platforms. Moreover, immune responses vary significantly depending on host species, breed, and physiological status, complicating the standardization of immunization protocols across different livestock populations [42,65]. Recent meta-analyses underscore the importance of tailoring vaccine formulations and adjuvant systems to specific host–pathogen dynamics to achieve consistent protective efficacy and overcome inter-species variability [43,65].

One of the most serious challenges remains the DIVA problem (Differentiating Infected from Vaccinated Animals). The ability to distinguish vaccinated from naturally infected animals is crucial for brucellosis control and eradication. However, current vaccines do not provide such properties, complicating seromonitoring and implementation of international eradication programs [66].

Thus, modern challenges in brucellosis vaccine development are related to the need to achieve a balance between safety and efficacy, ensuring long-term immunity, solving the DIVA problem, and reducing production and logistical barriers. Despite progress in creating recombinant and vector platforms, a universal vaccine satisfying all specified criteria does not yet exist. Promising directions remain the search for optimal antigen combinations, development of innovative adjuvants and delivery systems, and creation of economically accessible and thermostable preparations capable of ensuring global brucellosis control [32,65].

When developing brucellosis vaccines, it is important to consider multiple factors: first, how to obtain approval; second, to evaluate vaccine efficacy in two experimental animals, mice and monkeys; and finally, to test the vaccine for safety, immunogenicity, and efficacy. Preventive measures should have an effect even if it is impossible to test and demonstrate efficacy in humans [46].

## 7. Promising Directions and New Technologies in Brucellosis Vaccine Development

Modern achievements in molecular biology, genetic engineering, and nanotechnology open new possibilities for developing safer, more effective, and DIVA-compatible vaccines against brucellosis. In recent years, researchers’ attention has focused on several innovative approaches that can overcome existing limitations of traditional vaccines and ensure the development of a robust and specific immune response in small ruminants [43,66]. Modern approaches in reverse vaccinology and CRISPR-mediated genome-wide screening have significantly accelerated the identification of evolutionarily conserved protective antigens. This progress establishes a scientific foundation for the rational design of multicomponent vaccine platforms compatible with DIVA diagnostics and characterized by optimized safety profiles [66,67].

mRNA Vaccines. Development of mRNA vaccines, which demonstrated high efficacy in combating viral infections in humans, is increasingly being considered in veterinary medicine. Their key advantages include high creation speed, flexibility in antigen selection, and the possibility of inducing both humoral and cellular Th1 responses necessary for protection against *Brucella*. Moreover, mRNA platforms are potentially compatible with the DIVA principle, making them promising for use in disease control and eradication programs. The main challenge remains ensuring mRNA vaccine stability and adapting production technologies to agricultural application conditions [68].

Nanoparticles and Innovative Delivery Systems. Using nanoparticles, liposomes, virus-like particles, and polymeric carriers allows for significantly improving antigen presentation efficiency to the immune system. Such systems ensure prolonged release, targeted delivery to antigen-presenting cells, and enhancement of cellular immune response. An additional advantage is the possibility of creating thermostable forms less dependent on the cold chain, which is particularly important for application in endemic regions with limited infrastructure [69].

CRISPR/Cas9 and Genetic Engineering. Genome editing technologies, including CRISPR/Cas9, provide new tools for creating attenuated *Brucella* strains with controlled virulence and immunogenicity profiles. This approach allows targeted exclusion of pathogenicity genes and construction of vaccine strains with enhanced safety. Moreover, reverse vaccinology and bioinformatics analysis methods using CRISPR technologies are applied to identify new protective antigens, opening the way to creating multicomponent and next-generation recombinant vaccines [67].

Mucosal Vaccines and Intranasal Administration. Considering that *Brucella* penetrates through mucous membranes of respiratory and digestive tracts, developing mucosal vaccines is a priority direction. Intranasal and oral forms are capable of forming both systemic and local immunity at infection entry gates, providing an additional level of protection. A promising solution is using probiotic microorganisms, such as Lactobacillus or Bifidobacterium, as *Brucella* antigen carriers, which combines immunization with beneficial effects on microbiota [70].

“One Health” Approach and Interdisciplinary Research. Development of new brucellosis vaccines should be considered in the context of the “One Health” concept, uniting veterinary, medical, environmental, and social aspects of infection control. Successful implementation of vaccine strategies requires integration of molecular biotechnologies, immunology, epidemiology, and health economics. Interdisciplinary research allows for creating not only innovative preparations but also effective programs for their implementation, ensuring long-term control of zoonotic infection.

Despite the clear benefits of the One Health framework, its practical implementation is often hindered by institutional fragmentation, limited cross-sectoral data sharing, and disparities in funding between veterinary and public health systems. Strengthening interagency communication, harmonizing surveillance protocols, and establishing joint veterinary-medical task forces are essential steps toward sustainable brucellosis control in endemic regions.

Human-oriented vaccine platforms. Among the most promising candidates for future human brucellosis vaccines are subunit, DNA, and vector-based platforms currently undergoing preclinical evaluation. These approaches offer advantages in safety, DIVA compatibility, and the ability to target conserved *Brucella* antigens relevant across species. While clinical data in humans remain scarce, ongoing research in this direction underscores the translational relevance of veterinary findings and supports continued investment in cross-species vaccine development under the One Health framework.

Multicomponent delivery via innovative platforms. A key advantage of vector-based (e.g., Flu-BA), mRNA, and nanoparticle platforms is their capacity to co-deliver multiple *Brucella* antigens within a single formulation. This approach addresses the limitation of monocomponent vaccines while simplifying production, quality control, and field application compared to traditional multivalent preparations. Such platforms thus represent a pragmatic path forward for developing efficacious, DIVA-compatible brucellosis vaccines.

## 8. Limitations of the Review

This review has several methodological limitations that should be acknowledged. The literature search was restricted to three international databases (PubMed, Scopus, and Web of Science) and peer-reviewed articles published in English between 1990 and 2025. While this approach ensured high methodological standards and reproducibility, it may have excluded regionally published studies, dissertations, national veterinary reports, and other grey literature sources, particularly from brucellosis-endemic regions in Central Asia, the Middle East, and Africa. This could potentially affect the geographical representativeness of the epidemiological data synthesized in this review.

To mitigate this risk, we additionally analyzed official reports from the World Health Organization (WHO) and the World Organisation for Animal Health (WOAH), and incorporated verified epidemiological data from the Republic of Kazakhstan based on publications available in international indexes. Furthermore, future updates of this review should consider expanding the search strategy to include specialized veterinary databases (e.g., CAB Abstracts, AGRIS) and regional scientific platforms, provided that resources are available for critical appraisal and translation of non-English materials.

Translational considerations. Immunobiological differences between species—including variations in MHC haplotypes, cytokine profiles, and intracellular trafficking of *Brucella*—may limit direct application of veterinary vaccine data to human contexts. Consequently, protective efficacy observed in livestock or rodent models should be interpreted as indicative rather than definitive for human vaccine development. This review acknowledges these constraints and emphasizes the need for dedicated preclinical studies in relevant humanized models before clinical translation.

## 9. Conclusions

Analysis of modern achievements in brucellosis vaccine development demonstrates that, despite the availability of live attenuated preparations (e.g., *B. melitensis* Rev.1), they do not meet modern safety and epidemiological control requirements. Main challenges include the risk of abortions in pregnant animals, residual virulence, and inability to differentiate vaccinated from infected individuals (DIVA problem). Meanwhile, vector and recombinant platforms, particularly those based on influenza virus (Flu-BA), have shown high promise, combining immunogenicity, protective efficacy, and a more favorable safety profile.

Recommendations for future research include integration of advanced technologies (mRNA platforms, nanoparticles, targeted delivery systems, and CRISPR/Cas9 tools for optimizing antigen constructs), development of mucosal vaccines and alternative administration routes (intranasal and oral) to improve application convenience and immune response efficacy, conducting multicenter studies considering breed and age characteristics of animals, and strengthening interdisciplinary interaction within the “One Health” concept to reduce zoonotic risks to humans.

It is important to distinguish between vaccine strategies that have demonstrated proven efficacy in livestock and those with translational potential for human application. While live attenuated vaccines (e.g., *B. abortus* S19, *B. melitensis* Rev.1) remain the cornerstone of veterinary brucellosis control, their residual pathogenicity and DIVA incompatibility limit direct application in humans. Emerging platforms—including vector-based (Flu-BA), subunit, mRNA, and DNA vaccines—offer promising avenues for future human vaccine development, provided that dedicated preclinical studies in relevant models and early-phase clinical trials validate their safety and immunogenicity profiles.

Since no approved vaccine preventing brucellosis in humans exists, animal vaccination has become an important part of combating human brucellosis. Generally, live attenuated vaccines, widely known as *B. abortus* S19 and *B. melitensis* Rev.1 strains, are one of the most popular methods of animal immunization worldwide against brucellosis. Nevertheless, they have several disadvantages, including causing abortions in pregnant animals, pathogenicity for humans, development of antibodies to *Brucella* that hinder serological diagnosis of brucellosis, and resistance to antibiotics used for brucellosis treatment. Currently, there is no vaccine against human brucellosis. In this regard, vaccination against brucellosis in animals has made a significant contribution to combating human brucellosis.

The absence of a licensed human brucellosis vaccine remains a critical gap, constrained by ethical limitations on clinical trials, safety concerns with traditional live strains, and limited commercial incentives. Bridging this gap will require coordinated international efforts, increased investment in translational research, and accelerated development of next-generation platforms (subunit, mRNA, and viral vectors) that can safely induce durable cellular immunity in humans. Until such vaccines become available, robust veterinary immunization programs remain the cornerstone of human brucellosis prevention.

## Data Availability

No new data were created or analyzed in this study. Data sharing is not applicable to this article.

## References

[1] Wang W., Lu S., Li S., Ree M.J., Cui W. (2020). A man with recurrent fever, arthritis, and rash—*Brucellosis*? A case report. BMC Infect. Dis..

[2] Elbehery A., Aldubaib M., Al Rugaie O., Marzouk E., Abaalhail M., Moussa I., El-Husseiny M.H., Abaalhail A., Rawway M. (2022). Proteomic screening and antibiotic resistance assessment of clinical and subclinical *Brucella* species: Evolution of *Brucellosis* infection control. PLoS ONE.

[3] Elbehery A., Aldubaib M., Al Rugaie O., Marzouk E., Moussa I., El-Husseiny M., Ibrahim M., Abalhail A., Rawway M. (2022). *Brucella* species-induced brucellosis: Antimicrobial effects, potential resistance, and toxicity of silver and gold nanoparticles. PLoS ONE.

[4] Laine C.G., Johnson V.E., Scott H., Arenas-Gamboa A.M. (2023). Global Estimate of Human *Brucellosis* Incidence. Emerg. Infect. Dis..

[5] Centers for Disease Control and Prevention (2023). CDC Estimates Human *Brucella* Infections Could be Four Times Higher Than Previously Thought. Food Safety.

[6] Dereje T., Benti D., Feyisa B., Abiy G. (2018). Review of common causes of abortion in dairy cattle in Ethiopia. J. Vet. Med. Anim. Health.

[7] El-Diasty M., El-Said R., Abdelhalek A. (2021). Seroprevalence and molecular diagnosis of sheep *Brucellosis* in Dakahlia province, Egypt. Ger. J. Vet. Res..

[8] Dione M.M., Séry A., Sidibé C.A.K., Wieland B., Fall A. (2022). Exposure to multiple pathogens—Serological evidence of Rift Valley fever virus, *Coxiella burnetii*, bluetongue virus, and *Brucella* spp. in cattle, sheep, and goats in Mali. PLoS Negl. Trop. Dis..

[9] Dadar M., Shahali Y., Whatmore A.M. (2018). Human *Brucellosis* caused by raw dairy products: A review of prevalence, major risk factors, and prevention. Int. J. Food Microbiol..

[10] Maichomo M.W., McDermott J.J., Arimi S.M., Gathura P.B., Mugambi T.J., Muriuki S.M. (2007). Study of *Brucellosis* in a pastoral community and evaluation of the usefulness of clinical signs and symptoms in differentiating it from other flu-like diseases. Afr. J. Health Sci..

[11] Franco M.P., Mulder M., Smits H.L. (2007). Persistence and relapse in *Brucellosis* and need for improved treatment. Trans. R. Soc. Trop. Med. Hyg..

[12] Rossetti C.A., Arenas-Gamboa A.M., Maurizio E. (2017). Caprine *Brucellosis*: A historically neglected disease with significant impact on public health. PLoS Negl. Trop. Dis..

[13] Bosilkovski M., Keramat F., Arapović J. (2021). Current therapeutic strategies in human *Brucellosis*. Infection.

[14] Barakat A. (2025). Brucellosis. Medscape.

[15] World Health Organization (2020). Brucellosis. WHO Fact Sheets.

[16] Khurana S.K., Sehrawat A., Tiwari R., Prasad M., Gulati B., Shabbir M.Z., Chhabra R., Karthik K., Patel S.K., Pathak M. (2021). *Bovine brucellosis*—A comprehensive review. Vet. Q..

[17] Pal M., Gizaw F., Fekadu G., Alemayehu G., Kandi V. (2017). Public health and economic importance of *Bovine brucellosis*: An overview. Am. J. Epidemiol. Infect. Dis..

[18] Charypkhan D., Sultanov A.A., Ivanov N.P., Baramova S.A., Taitubaev M.K., Torgerson P.R. (2019). Economic and Medical Burden of Brucellosis in Kazakhstan. Zoonoses Public Health.

[19] Kassymov E., Isabaev A., Oshakbaeva N., Aubakirov M., Erenko E., Raketsky V., Sharipova A. (2025). Monitoring of the Epizootic Situation of Brucellosis among Animals and Humans in Kostanay Region. Nauka Obraz..

[20] Shevtsova E., Shevtsov A., Mukanov K., Filipenko M., Kamalova D., Sytnik I., Syzdykov M., Kuznetsov A., Akhmetova A., Zharova M. (2016). Epidemiology of *Brucellosis* and Genetic Diversity of *Brucella abortus* in Kazakhstan. PLoS ONE.

[21] Ivanov N.P. (2007). Brucellosis in Animals and Control Measures.

[22] Akpinar O. (2016). Historical perspective of *Brucellosis*: A microbiological and epidemiological overview. Infez. Med..

[23] Moreno E., Moriyón I. (2002). *Brucella melitensis*: A nasty bug with hidden credentials for virulence. Proc. Natl. Acad. Sci. USA.

[24] Young E.J., Mandell G.L., Bennett J.E., Dolin R. (2005). Brucella Species. Principles and Practice of Infectious Diseases.

[25] Godfroid J., Käsbohrer A. (2002). Brucellosis in the European Union and Norway at the Turn of the Twenty-First Century. Vet. Microbiol..

[26] Liautard J.P., Gross A., Dornand J., Köhler S. (1996). Interactions between professional phagocytes and *Brucella* spp.. Microbiology.

[27] Barquero-Calvo E., Chaves-Olarte E., Weiss D.S., Guzman-Verri C., Chacon-Diaz C., Rucavado A., Moriyón I., Moreno E. (2007). *Brucella abortus* uses a stealthy strategy to avoid activation of the innate immune system during the onset of infection. PLoS ONE.

[28] Martirosyan A., Moreno E., Gorvel J.P. (2011). An evolutionary strategy for a stealthy intracellular *Brucella* pathogen. Immunol. Rev..

[29] Refai M. (2002). Incidence and control of *Brucellosis* in the Near East region. Vet. Microbiol..

[30] Kiros A., Asgedom H., Abdi R.D. (2016). A review on bovine *Brucellosis*: Epidemiology, diagnosis, and Control Options. ARC J. Anim. Vet. Sci..

[31] Li Z.Q., Shi J.X., Fu W.D., Zhang Y., Zhang J., Wang Z., Li T.-S., Chen C.-F., Guo F., Zhang H. (2015). A *Brucella melitensis* M5-90 wboA deletion strain is attenuated and enhances vaccine efficacy. Mol. Immunol..

[32] Ducrotoy M.J., Bertu W.J., Matope G., Cadmus S., Conde-Alvarez R., Gusi A.M., Welburn S., Ocholi R., Blasco J.M., Moriyón I. (2017). *Brucellosis* in Sub-Saharan Africa: Current challenges for management, diagnosis and control. Acta Trop..

[33] Hou H., Liu S., Peng C. (2019). The advances in *Brucellosis* vaccines. Vaccine.

[34] Dorneles E.M., Sriranganathan N., Lage A.P. (2015). Recent Advances in *Brucella abortus* Vaccines. Vet. Res..

[35] Ko J., Splitter G.A. (2003). Molecular host-pathogen interaction in *Brucellosis*: Current understanding and future approaches to vaccine development for mice and humans. Clin. Microbiol. Rev..

[36] Lalsiamthara J., Lee J.H. (2017). Development and trial of vaccines against *Brucella*. J. Vet. Sci..

[37] Kim W.K., Moon J.Y., Cho J.S., Ochirkhuyag E., Akanda M.R., Park B.Y., Hur J. (2019). Protective efficacy of an inactivated *Brucella abortus* vaccine candidate lysed by GI24 against *Brucellosis* in Korean black goats. Can. J. Vet. Res..

[38] Dzata G.K., Confer A.W., Wyckoff J.H. (1991). The effects of adjuvants on immune responses in cattle injected with a *Brucella abortus* soluble antigen. Vet. Microbiol..

[39] Blasco J.M., Gamazo C., Winter A.J., Jiménez de Bagüés M.P., Marín C., Barberán M., Moriyón I., Alonso-Urmeneta B., Díaz R. (1993). Evaluation of whole cell and subcellular vaccines against *Brucella ovis* in rams. Vet. Immunol. Immunopathol..

[40] Tabatabai L.B., Pugh G.W., Stevens M.G., Phillips M., McDonald T.J. (1992). Monophosphoryl lipid A-induced immune enhancement of *Brucella abortus* salt-extractable protein and lipopolysaccharide vaccines in BALB/c mice. Am. J. Vet. Res..

[41] Ranjbar R., Sharifimoghadam S., Saeidi E., Jonaidi N., Sheikhshahrokh A. (2016). Induction of protective immune responses in mice by double DNA vaccine encoding of *Brucella melitensis* Omp31 and *Escherichia coli* Eae genes. Trop. J. Pharm. Res..

[42] Martirosyan A., Gorvel J.P. (2013). *Brucella* Evasion of Adaptive Immunity. Future Microbiol..

[43] Ingolotti M., Kawalekar O., Shedlock D.J., Muthumani K., Weiner D.B. (2010). DNA vaccines for targeting bacterial infections. Expert Rev. Vaccines.

[44] Al-Mariri A., Tibor A., Lestrate P., Mertens P., De Bolle X., Letesson J.-J. (2002). Yersinia enterocolitica as a vehicle for a naked DNA vaccine encoding *Brucella abortus* bacterioferritin or P39 antigen. Infect. Immun..

[45] Singha H., Mallick A.I., Jana C., Fatima N., Owais M., Chaudhuri P. (2011). Co-immunization with interleukin-18 enhances the protective efficacy of liposomes encapsulated recombinant Cu–Zn superoxide dismutase protein against *Brucella abortus*. Vaccine.

[46] Perkins S.D., Smither S.J., Atkins H.S. (2010). Towards a *Brucella* vaccine for humans. FEMS Microbiol. Rev..

[47] Ficht T.A., Kahl-McDonagh M.M., Arenas-Gamboa A.M., Rice-Ficht A.C. (2009). *Brucellosis*: The case for live, attenuated vaccines. Vaccine.

[48] Tabynov K. (2016). Influenza viral vector-based *Brucella abortus* vaccine: A novel vaccine candidate for veterinary practice. Expert Rev. Vaccines.

[49] Ryskeldinova S., Zinina N., Kydyrbayev Z., Yespembetov B., Kozhamkulov Y., Inkarbekov D., Assanzhanova N., Mailybayeva A., Bugybayeva D., Sarmykova M. (2021). Registered Influenza Viral Vector-Based *Brucella abortus* Vaccine for Cattle in Kazakhstan: Age-wise safety and Efficacy Studies. Front. Cell. Infect. Microbiol..

[50] Tabynov K., Sansyzbay A., Kydyrbayev Z., Yespembetov B., Ryskeldinova S., Zinina N., Egorov A. (2014). Influenza viral vectors expressing the *Brucella* Omp16 or L7/L12 proteins as vaccines against *B. abortus* infection. Virol. J..

[51] Tabynov K., Kydyrbayev Z., Ryskeldinova S., Yespembetov B., Zinina N., Assanzhanova N., Sansyzbay A. (2014). Novel influenza virus vectors expressing *Brucella* L7/L12 or Omp16 proteins in cattle induced a strong T-cell immune response, as well as high protectiveness against *B. abortus* infection. Vaccine.

[52] Tabynov K., Yespembetov B., Ryskeldinova S., Zinina N., Kydyrbayev Z., Kozhamkulov Y., Sansyzbay A. (2016). Prime-booster vaccination of cattle with an influenza viral vector *Brucella abortus* vaccine induces a long-term protective immune response against *Brucella abortus* infection. Vaccine.

[53] Mailybayeva A., Yespembetov B., Ryskeldinova S., Zinina N., Sansyzbay A., Renukaradhya G.J., Tabynov K. (2017). Improved influenza viral vector-based *Brucella abortus* vaccine induces robust B and T-cell responses and protection against *Brucella melitensis* infection in pregnant sheep and goats. PLoS ONE.

[54] Bugybayeva D., Ryskeldinova S., Zinina N., Sarmykova M., Assanzhanova N., Kydyrbayev Z., Tabynov K. (2020). Development of human vectored *Brucellosis* Vaccine Formulation: Assessment of Safety and Protectiveness of Influenza Viral Vectors Expressing *Brucella* Immunodominant Proteins in Mice and Guinea Pigs. Biomed. Res. Int..

[55] Bugybayeva D., Kydyrbayev Z., Zinina N., Assanzhanova N., Yespembetov B., Kozhamkulov Y., Zakarya K., Ryskeldinova S., Tabynov K. (2021). A new candidate vaccine for human *Brucellosis* based on influenza viral vectors: A preliminary investigation for the development of an immunization schedule in a guinea pig model. Infect. Dis. Poverty.

[56] Sharif F., Nazari R., Fasihi-Ramandi M., Taheri R.A., Zargar M. (2024). Intranasal and intraperitoneal immunization against *Brucella* infection using niosome and mannosylated niosomes containing *Brucella* recombinant trigger factor/Bp26/Omp31 chimeric protein in a mouse model. Clin. Exp. Vaccine Res..

[57] Gheibi A., Khanahmad H., Kashfi K., Sarmadi M., Khorramizadeh M.R. (2018). Development of new generation of vaccines for *Brucella abortus*. Heliyon.

[58] Hu X.D., Yu D.H., Chen S.T., Li S.-X., Cai H. (2009). A combined DNA vaccine provides protective immunity against Mycobacterium bovis and *Brucella abortus* in cattle. DNA Cell Biol..

[59] Chen B., Liu B., Zhao Z., Wang G. (2019). Evaluation of a DNA vaccine encoding *Brucella* BvrR in BALB/c mice. Mol. Med. Rep..

[60] Shojaei M., Tahmoorespur M., Soltani M., Sekhavati M.H. (2018). Immunogenicity evaluation of plasmids encoding *Brucella melitensis* Omp25 and Omp31 antigens in BALB/c mice. Iran. J. Basic Med. Sci..

[61] Imtiaz W., Khan A., Gul S.T., Saqib M., Saleemi M.K., Shahzad A., Dong J., Hussain R., Shen M., Du X. (2018). Evaluation of DNA vaccine encoding BCSP31 surface protein of *Brucella abortus* for protective immunity. Microb. Pathog..

[62] Al-Mariri A., Abbady A.Q. (2013). Evaluation of the immunogenicity and the protective efficacy in mice of a DNA vaccine encoding SP41 from *Brucella melitensis*. J. Infect. Dev. Ctries..

[63] Jain S., Afley P., Dohre S.K., Saxena N., Kumar S. (2014). Evaluation of immunogenicity and protective efficacy of a plasmid DNA vaccine encoding ribosomal protein L9 of *Brucella abortus* in BALB/c mice. Vaccine.

[64] Darbandi A., Alamdari S.Z., Kupai M., Ganavati R., Heidari M., Talebi M. (2022). Evaluation of immune responses to *Brucella* vaccines in mouse models: A systematic review. Front. Vet. Sci..

[65] Li L.M., Xiang W.T., Li T., Xiang M.M., Liu F., Li J.M. (2024). Efficacy of *Brucella* vaccines in sheep: A systematic review and meta-analysis. Transbound. Emerg. Dis..

[66] Yang X., Skyberg J.A., Cao L., Clapp B., Thornburg T., Pascual D.W. (2013). Progress in *Brucella* vaccine development. Front. Biol..

[67] Xu H., Lu J., Huang F., Zhang Q., Liu S., Chen Z., Li S. (2024). A genome-wide CRISPR screen identified host genes essential for intracellular *Brucella* survival. Microbiol. Spectr..

[68] Luo J.R., Qi S.H., Tian T.T., Shang K.-Y., Shi H.-D., Li C., Chai Z.-L., Ding J.-B., Zhu Y.-J., Zhang F.-B. (2025). Design of a multi-epitope mRNA vaccine against the *Brucella* type IV secretion system using reverse vaccinology and immunogenicity approaches. Sci. Rep..

[69] Sadeghi Z., Fasihi-Ramandi M., Bouzari S. (2020). Nanoparticle-based vaccines for *Brucellosis*: Calcium phosphate nanoparticles-adsorbed antigens induce cross-protective response in mice. Int. J. Nanomed..

[70] Rollier C.S., Reyes-Sandoval A., Cottingham M.G., Ewer K., Hill A.V.S. (2011). Viral Vectors as Vaccine Platforms: Deployment in Sight. Curr. Opin. Immunol..

